# The Effects of Video Visual Scene Displays on the Symbolic Communication of Preschool Children with Neurodevelopmental Disabilities and Their Peers in a Shared Video Activity

**DOI:** 10.3390/bs16060935

**Published:** 2026-06-06

**Authors:** Dana Patenaude, David McNaughton, Rebecca DeLaMare

**Affiliations:** 1 School of Inclusive Teacher Education, Bowling Green State University, Bowling Green, OH 43403, USA; 2 Department of Educational Psychology, Counseling, and Special Education, Pennsylvania State University, University Park, PA 16802, USA; dbm2@psu.edu (D.M.); rdw5458@psu.edu (R.D.)

**Keywords:** preschoolers, neurodevelopmental disabilities, autism, peer interactions, augmentative and alternative communication

## Abstract

Many children who experience speech and communication disabilities use or could benefit from the use of augmentative and alternative communication (AAC). Despite the potential benefits of AAC use for these children, many experience challenges in using traditional AAC systems to communicate with others, including their peers. Without effective peer interactions, children can miss out on valuable opportunities for social interaction and the development of communication skills. Video visual scene displays (VSDs) offer a unique alternative to support children in engaging in social communication. Video VSDs embed videos, a preferred activity for many children, with relevant vocabulary that is programmed as hotspots to support communication. The purpose of this study was to examine the impacts of a video VSD approach on the symbolic communication of preschool children with neurodevelopmental disabilities and delays (including autism) during a shared high-interest video activity with neurotypical peers. All three children showed increased symbolic communication frequency, with two demonstrating very large effect sizes and one a moderate effect. Future research should evaluate the use of video VSDs in a shared video activity with decreased researcher involvement and should examine change in participants’ vocabulary as a result of the video VSD activity.

## 1. Introduction

### 1.1. Peer Interaction

Peer social interaction provides critically important opportunities for children to enjoy friendships, learn social interaction routines, and acquire language and communication skills (Biggs et al., 2025; Kasari et al., 2011; Laubscher et al., 2022b; Therrien & Light, 2016, 2018). Children typically begin to show an interest in interacting with peers by age 3, and peer interaction continues to grow in importance across the lifespan for all children (Carter et al., 2010, 2015; Odom et al., 1999). For preschool-aged children, peer social interaction has been found to influence language growth, the development of identity, and the quantity and quality of friendships (Chen et al., 2020; Ladd & Coleman, 1997; Stanton-Chapman & Snell, 2011).

For children with neurodevelopmental disabilities (NDD), including autism, Down syndrome and other disabilities, participating in social exchanges with peers may be challenging (Bowman-Perrott et al., 2023). Many children with NDD experience difficulty with speech, which may have negative impacts on their ability to interact with both peers and adults. For example, speech will not meet the communication needs of approximately 25–30% of autistic children at age 5 (Tager-Flusberg & Kasari, 2013). Without appropriate communication supports for social interaction, children with speech-related disabilities are at a higher risk for negative outcomes in language acquisition, cognitive development, academic participation, and the formation of friendships (Blasko, 2025; Chen et al., 2020; Ganz, 2015; Light et al., 2025). 

In response to this challenge, a growing body of research has investigated interventions to support the social interaction of children with NDD with their typically developing peers in early childhood settings (Biggs et al., 2025; S. E. Chapin et al., 2022; Laubscher et al., 2022a; Severini et al., 2019). The research to date highlights the importance of three areas: communication supports for the child with NDD (Boyle et al., 2017, 2021; Laubscher et al., 2019; Therrien & Light, 2018); the use of age-appropriate and motivating activities (S. E. Chapin et al., 2022; Lorah et al., 2014; Therrien & Light, 2018; Trembath et al., 2009); and the provision of easily learned and implemented interaction supports for both the child with NDD and their neurotypical peers (Ganz & Flores, 2008; Holyfield et al., 2025; Lorah et al., 2014). 

### 1.2. Communication Supports

Augmentative and alternative communication (AAC) can be an effective communication support for individuals who do not consistently or reliably make use of spoken language to communicate (Biggs et al., 2018; Morin et al., 2018). However, traditional forms of AAC can be difficult for children to learn and use. For example, traditional grid displays require the AAC user to triangulate attention between the communication device, the communication partner, and the activity (Light et al., 2019; J. McCarthy & Boster, 2019). Additionally, commonly used representations, such as line drawings, can be challenging for young children to interpret and use (Worah et al., 2015; J. W. McCarthy et al., 2018). 

Perhaps most significantly, many past AAC interventions have focused on the expression of needs and wants (Iacono et al., 2016; Light & McNaughton, 2015; Morin et al., 2018). Although requesting is an important skill, communication intervention must also address the wide variety of skills used in social interaction such as commenting, asking questions, and expressing preferences (Corbin, 2025; Holyfield et al., 2025; Zimmerman, 2025). Despite some recent work (Severini et al., 2019; Therrien & Light, 2016, 2018), there has been limited attention to supporting social interaction between children with NDD who could benefit from AAC and their neurotypical peers within preschool settings. 

### 1.3. High-Interest Activities

In order to create engaging and potentially impactful interventions, both the activity used and the vocabulary available as communication supports should be of interest to the children. The incorporation of child interests into instruction has been found to be associated with increased motivation and positive outcomes for young children (Fedewa et al., 2024). Introducing and supporting the use of vocabulary for preferred topics is critical because children are most likely to learn language and communication skills during these high-interest interactions (Harrop et al., 2019; Koegel et al., 2012; Watkins et al., 2019). Traditionally, in peer support interventions, the materials used are most commonly selected based on the preferences of the children with NDD or typical classroom routines (S. Chapin et al., 2018). This may promote the engagement of the children with NDD; however, the neurotypical peer participants may not have their interests reflected in the instruction. Incorporating the interests of all children in the interaction, with or without NDD, and may increase interest and engagement in peer interactions.

### 1.4. Supports for Social Interaction

Traditional interventions to promote interactions between children with NDD who would benefit from the use of AAC and their neurotypical peers often require training for the peer to be an effective communication partner (S. Chapin et al., 2018; Thiemann-Bourque, 2012). Though training neurotypical peers to be communication partners has been found to contribute to positive social communication gains for children with NDD, extensive peer training may be inaccessible or inappropriate for peer social relationship development. Training a peer to create successful communication opportunities for a child with NDD may contribute to power imbalance in social relationships between children with and without NDD (Biggs et al., 2025; Therrien et al., 2016). Additionally, peer trainings often involve several sessions with multiple steps. The resources necessary to train peers could pose a challenge in a typical classroom environment (Chan et al., 2009; Chang & Locke, 2016). Finally, in peer-mediated interventions, often only a select few peers who have formal, individualized trainings are interacting with children with NDD within the context of the research study. Interaction supports should be able to be quickly and easily used by peers in order to alleviate challenges with extensive training procedures. 

To effectively support children with NDD in increasing participation in social communication, intervention research must address three goals: (a) provide communication supports that can be efficiently and successfully used by preschool children with NDD, (b) use activities that incorporate the interests of children with and without NDD, and (c) provide interaction supports that are both quickly learned and promote equitable roles for both the neurotypical peers and the children with NDD.

### 1.5. Visual Scene Displays

The use of a video visual scene display (VSD) may offer a promising approach to addressing these three major goals. VSDs are contextualized images (e.g., photographs, stills from a video) that can be used to support communication for individuals who need or use AAC. VSDs are created by uploading images to VSD applications housed on tablet computers and then using the VSD app to program meaningful vocabulary within areas (i.e., “hotspots”) that when activated, produce speech or sounds (Light et al., 2019). A video VSD approach combines both videos and VSDs. A high-interest video plays for a period of time, and then pauses to display a VSD (i.e., a still image with programmed hotspots). VSDs address the common challenges of traditional AAC systems by adding contextual images, meaningful vocabulary, and decreasing the need for the user to triangulate their attention (Holyfield et al., 2025; J. McCarthy & Boster, 2019).

VSDs have been identified as an evidence-based practice for increasing the communication of young children with speech-related disabilities (Patenaude et al., 2024). Previous work on VSDs has focused on their use for increasing single-word reading and communication skills using e-books between children with disabilities (i.e., autism, developmental delays, Down syndrome) and their peers (Boyle et al., 2021), and in shared storybook reading (Therrien & Light, 2016, 2018). Boyle and colleagues found that with the use of a VSD e-book, all participants increased their single-word reading. Therrien and Light (2016; 2018) found that all autistic participants increased their communication with the use of VSDs during shared storybook activities. VSDs have demonstrated effectiveness across a range of instructional activities and with children diagnosed with various disabilities.

More recently, video VSDs have been used to increase communicative turn-taking by autistic preschool-aged children (S. E. Chapin et al., 2022). This is a promising area for research, as although videos are commonly identified as a highly preferred activity for both children with NDD as well as children without NDD, the watching of videos typically involves very limited interactions with others (Kucker et al., 2024). As a first step towards investigating the potential benefits of a video VSD approach with preschoolers who need or use AAC, Chapin and colleagues assessed the impact of highly preferred videos programmed with meaningful vocabulary on the communication of autistic preschool children (2022). They reported that all three participants demonstrated increases in the number of communicative acts with the use of video VSDs. It is important to note, however, that video VSD sessions were conducted by the researcher with the participants, and no peers were involved in these interactions. 

### 1.6. Current Study

In summary, there is growing evidence to suggest that VSDs support peer interactions, and video VSDs appear to be a promising approach to supporting communication for children with NDD. Video VSDs can be easily learned and used by children with NDD (S. E. Chapin et al., 2022). VSDs and video VSDs can also be easily created to support the interests of the child participants (Holyfield et al., 2025; Laubscher et al., 2022b). Finally, the inclusion of hotspots within the video provides a natural support for interaction and turn-taking, reducing the need for instruction for the child with NDD and their peer (Babb et al., 2021; S. E. Chapin et al., 2022).

To date, no research has been conducted on the potential benefits of video VSDs in a peer interaction involving preschool children with and without NDD. This study therefore addressed the following questions:

1. What are the effects of video VSDs (and brief instruction in their use) on the number of symbolic communicative acts of preschool children with NDD during a shared video-watching activity with peers without NDD?
2. What are the perceptions of consumers (i.e., teachers, participants) regarding the intervention?

## 2. Materials and Methods

Positionality and reflexivity statements (i.e., statements explaining the authors’ affiliations and experiences to explain potential biases), though prevalent in qualitative research, are growing in popularity in quantitative research (Jamieson et al., 2023). In this study, the first author is a white woman who has served as a Board-Certified Behavior Analyst, supporting autistic children and their families to promote social communicative skills. She is also a researcher and professor of Special Education with a focus on social, emotional, and behavioral supports in schools in addition to assistive technology and AAC. The second author is a white man who works as a professor of Special Education, with a focus on literacy instruction and community support for people who need and use AAC. The third author is a white woman and a doctoral candidate in Special Education; she has served as a special educator for many years and is a mother of a child who used AAC.

### 2.1. Participants and Setting 

Participants were recruited from an inclusive preschool center in Pennsylvania. All study activities took place over the course of 11 weeks at the preschool center, which served approximately 55 children. The participants in this study included preschool children with diagnosed NDD, and also same-age peers without identified disabilities. The participants with NDD met the following criteria: (a) were between the ages of 3;0 and 5;11; (b) had been identified as having a neurodevelopmental delay or disability and were receiving services for a delay or disability at the center; (c) had vision, hearing, and motor abilities within normal limits according to parent report; (d) demonstrated interest in photographs and books; (e) resided in Pennsylvania; and (f) spoke English in the home. The neurotypical peer participants met the following criteria: (a) were between the ages of 3;0 and 5;11 years old; (b) had vision, hearing, and motor abilities within normal limits according to parent report; (c) did not have intellectual or developmental disabilities or delays based on parent report; (d) demonstrated interest in photographs and books; (e) resided in Pennsylvania; and (f) spoke English in the home. The study was approved by the Institutional Review Board of the affiliated university’s Office for Research Protections.

Four participants with NDD (Maggie, Beau, Dominic^1^, Carly) and six neurotypical participants (Bethany, Ellie, Harry, William, Kate, Kim) were recruited to participate in this study. Informed written consent was obtained from the parents of all the participants. The researcher obtained assent from the participants before each session by asking them if they would like to watch a video with a friend. The participants ranged in age from 3 years, 1 month to 5 years, 2 months at the time of the study. All four participants with NDD received speech language pathology services in their preschool setting. Refer to Table 1 for descriptions of the participants. 

### 2.2. Language Measures

To provide a more complete profile of the participants with NDD included in the study, the MacArthur-Bates Communicative Development Inventories (CDI; Fenson et al., 2007) was completed by a parent of each participant with NDD. The CDI is a norm-referenced assessment evaluating children on primary language skill areas such as vocabulary comprehension and production. A parent of each participant with NDD completed the Words and Gestures form; these forms were then reviewed by the researcher to develop an overview summary of language comprehension and production.

The researcher also utilized the Preschool Language Scales-5 Screener (PLS-5) to obtain screening level information regarding the current communicative development of all participants, with or without NDD. The PLS-5 Screener provides a norm-referenced criterion score that presents basic information on the language and speech skills of children between the ages of three and eight years old. The screener provides scores for six domains: language, articulation, connected speech, social/interpersonal, fluency, and voice. If the child does not pass the screener in any of these domains, the PLS-5 manual recommends obtaining additional information on that domain for that child. Because the PLS-5 is a screener and not a diagnostic assessment, the outcomes only indicate that the child should “receive further evaluation”. Additionally, given the constraints of data collection in an educational setting, and the fact that all children in this study already received comprehensive screening and assessment services as part of their participation in the early childhood program, the authors did not complete extensive language assessments.

Additionally, the researcher conducted a naturalistic language observation (approximately 90 min in total length) in the school setting. The researcher observed the participants with NDD engaging in instructional activities (e.g., circle time) and play time with peers to document the natural communication performance of the participants. The researcher also interviewed the teacher of each participant with NDD using language profile questions developed by the researcher about the participants’ communication. Topics addressed both expressive and receptive communication, including the participants’ ability to communicate socially and engage with others, request desired objects, and engage in social etiquette routines (e.g., greetings), as well as their ability to respond to their name and engage in joint attention. The information detailing the participants below was informed by the author’s own naturalistic observations and by interviews with parents, teachers, and speech and language pathologists. See Patenaude (2025) to view the teacher interview questions.

### 2.3. Participants

#### 2.3.1. Beau

Beau was four years, three months old at the time of the study. He was identified by his preschool speech language pathologist (SLP) as having a developmental delay impacting his social communication and language. Beau’s mother reported that Beau enjoyed coloring, dancing, and playing on the playground. In class, the researcher observed Beau following one- and two-step directions during structured activities (e.g., circle time). Beau was observed to primarily play solitarily and was not observed to spontaneously communicate with peers or teachers. Beau enjoyed engaging in sensory play and playing with toy cars. 

Beau’s teacher reported that Beau communicated using speech but often required prompting to do so. According to Beau’s CDI score, he understood 331 words and made use of 206 of those words. Beau’s PLS-5 scores indicated that he was observed to be within the range of typical performance for his chronological age in the articulation and voice domains, but he required further evaluation on language, connected speech, social/interpersonal, and fluency domains. Much of Beau’s speech was made up of one-to-two-word utterances that he produced in a quiet tone, accompanied by pointing to the referent. When he made use of speech, he often imitated the last word spoken to him by his communication partner, including peers, or produced unintelligible vocalizations. Beau was able to imitate one-to-two-word utterances but was not reported by teachers or observed by the researcher to initiate communication with others. At the time of the study, saying “yes” or “no” were the most frequently observed communication behaviors. Beau’s mother reported that when Beau was offered a preferred item, he repeated the word, appeared excited, and pointed to the item. Beau’s speech and language goals included increasing spontaneous communication and expanding the length of utterances (e.g., “want puzzle” rather than “puzzle”). 

To indicate that he did not like something being offered to him, or when asked to complete a non-preferred task, Beau would shake his head no. According to teacher report, he would physically remove himself from an activity, peer, or situation he did not like. Beau experienced challenges with sitting and attending to a non-preferred activity (e.g., circle time, academic instruction). He was reported to cry and throw a tantrum on occasion when directed to engage in non-preferred activities. At these times, his teachers would provide him with time and space with the goal of his returning to the activity at a later time.

#### 2.3.2. Maggie

Maggie was four years, eight months old at the time of the study. She was reported by school officials as having a neurological developmental disorder (Dandy–Walker Syndrome), which impacts various areas of development, including motor coordination and speech due to the underdevelopment of parts of the cerebellum (Sun et al., 2023). During free play opportunities at the preschool, Maggie enjoyed dramatic play, arts and crafts, and listening to books. With peers, Maggie was observed to seek out her peers for play activities, and frequently helped them in the classroom (e.g., guided friend to line up). She was able to engage in structured reciprocal interactions with peers (e.g., handing an item to a peer at circle time). However, when faced with peer conflict (e.g., having a toy taken), she had difficulty asserting herself or advocating for her needs. 

Maggie’s teacher reported that she made use of speech to request objects and activities and to label items in the classroom, typically in short phrases. According to Maggie’s CDI score, she understood 369 words and made expressive use of 363 of those words. On the PLS-5, Maggie scored within the range of typical performance for her chronological age in the social/interpersonal and voice domains, but she required further evaluation on language, articulation, connected speech, and fluency domains. She communicated her enjoyment of an activity (or frustration) by clenching her fists and bringing them up above her head. Maggie asked questions of her teachers and occasionally peers during the school day. For example, she would ask peers if she could take a turn with a toy during free play in the classroom. She was reported to be able to comment on activities in the classroom (e.g., express liking an activity), and would occasionally talk about events outside the classroom (e.g., describe something she had done at home). When peers or teachers would enter or exit the classroom, she would communicate greetings or closures. Maggie’s speech and communication goals included the expansion of spontaneous communication and interjections to advocate for herself with peers and teachers. Her teacher reported that she had increased her spontaneous communication since the start of the 2024–2025 school year. 

It was reported that Maggie often experienced difficulty in requesting assistance. At these times, she would stand in proximity to her teachers and either wait for them to ask her what she needed, or she would ask something unrelated to her current needs (e.g., “Can I have ChapStick?”). Some of Maggie’s speech was reported to be repetitive in nature. For example, Maggie was described as making use of a small number of phrases throughout the day, (e.g., saying that her lips hurt, or that her legs were itchy) and/or asking for confirmation of the next activity on the schedule (e.g., “Is it lunch now?”) at inappropriate times. 

Maggie would occasionally use short spoken phrases (e.g., “Me no like that”) to communicate that she did not like something. More frequently, she would sit at the activity and not engage or communicate her displeasure unless asked or prompted to continue the activity. For example, in activities in which the children would get messy (e.g., finger painting), Maggie would sit at the art table but would not participate in the activity unless prompted to do so, though she would not cry or refuse. Her teacher also reported that Maggie was very routine-based and struggled with changes to her routine (e.g., being dropped off to school late). 

#### 2.3.3. Carly 

Carly was five years, two months old at the time of the study. Carly received speech language pathology services both in and outside of her preschool setting due to a diagnosis of autism spectrum disorder and language delay. Carly also received behavior services within the preschool setting. In the classroom, Carly enjoyed engaging in dramatic play by herself, playing musical instruments (e.g., drums), and reading books. Carly was observed as an active child who enjoyed any physical movement play, including playing on the playground and running. Carly typically played by herself but would occasionally join peers in play activities when invited by her peers or teachers. 

Carly’s teacher reported that despite having a large vocabulary, Carly engaged in very limited spontaneous speech, though when prompted, she would speak in one-to-two-word utterances. According to Carly’s CDI score, she understood 386 words and made expressive use of 207 of those words. On the PLS-5, Carly scored within the typical range of performance in the voice domain, but she required further evaluation in the language, articulation, connected speech, social/interpersonal, and fluency domains. She infrequently communicated spontaneously with teachers, and very rarely with peers. When Carly liked or wanted something, she would express this by using speech (if prompted to use speech) or would take what she wanted (e.g., take a toy from a peer). Most of Carly’s communication was to request an item or label an item when asked to do so. Carly’s speech and language goals developed by her teacher and speech language pathologists included spontaneous communication and expansion of spoken utterances from one-word to two-word utterances. To do so, her educational staff utilized several visual supports, including first-then visuals and visuals with two empty boxes cueing two-word responses, to support her participation and communication.

Carly demonstrated her dislike of an activity (e.g., transitions, lesser preferred centers, peers taking toys) by engaging in self-injurious behaviors (e.g., head hitting), vocalizing loudly, and leaving the activity area. Carly was observed to have challenges with transitions to lesser preferred activities, such as circle time and leaving preferred centers (e.g., blocks and music). Additionally, structured academic tasks (e.g., writing) were non-preferred for Carly. 

#### 2.3.4. Dominic

Dominic was four years, nine months at the time of the study. He was identified as having a speech delay, along with specific challenges with social pragmatics as reported by his mother and his teacher. Because Dominic’s CDI and PLS-5 scores suggested that he had speech that was within typical developmental ranges, and based on observations, baseline data, and communication with his teacher and speech language pathologist, the video VSD intervention used in this study was determined to not be an appropriate strategy for supporting Dominic in meeting his communication and language goals. Given the nature of the informed consent signed by Dominic’s parents, the goal of this study (e.g., to evaluate changes in symbolic communication) did not meet an immediate communication need for Dominic and was therefore deemed inappropriate for him. Additionally, continuing consistent data collection sessions with Dominic would have resulted in reduced time in the classroom for him, where he was working on social communicative skill acquisition that met his needs. He was therefore withdrawn from further data collection after baseline sessions. After consulting with teachers and Dominic’s mother, the researcher continued intermittent sessions because Dominic enjoyed the video VSD activity, and because his teachers and parents believed the opportunities for structured peer engagements were beneficial to him.

### 2.4. Neurotypical Peers

The six neurotypical participants (Bethany, Ellie, Harry, William, Kate, Kim) ranged in age from 3 years, 1 month to 4 years, 8 months at the time of the study. The neurotypical participants were identified by parents as not having a neurodevelopmental disability and were reported to enjoy looking at photographs and watching videos. All the neurotypical peer participants passed all six domains on the PLS-5, which provides initial evidence that they all had language within the typical range of development for their age.

### 2.5. Materials

This study investigated the use of video VSDs as a communication support for children with NDD during researcher-supported dyadic interactions with peers without disabilities. All the videos were shown on a Samsung Galaxy Tab A8 Tablet^TM^^2^. The videos programmed in the video VSD app were selected based on the preferences of the children in the dyad.

#### 2.5.1. Identification of Preferences

The videos used in the dyadic interactions were developed based on the preferences of the child participants. The preferences of all nine child participants were identified with the use of a parent survey. All the parents were sent an open-ended survey (hosted on Google Forms) and were asked to identify ten or more activities (e.g., playing on the playground) and animals (e.g., pigs) that their child liked and/or disliked. The parents were also asked to identify two or more preferred and non-preferred YouTube videos (e.g., Cocomelon^3^, Baby Shark^4^).

#### 2.5.2. Compilation of Videos

Based on the preferences identified by parents, the researcher created 18 compilation videos. A total of six different compilation videos were created for each of the three participants with NDD (i.e., Beau, Maggie, Carly). Each compilation video was 6 min in duration and included twelve 30 s video clips of the parent-reported activities, animals, and YouTube videos. Short clips were used to provide a wide variety of engaging content for the participants. The 12 video clips were selected by the researcher so as to include six clips preferred or non-preferred by a specific participant with NDD (e.g., Beau), and six video clips selected from a pool of all preferred or non-preferred clips of the six neurotypical participants. The videos were created using Canva^5^^TM^, Adobe^6^^TM^ and YouTube^7^^TM^. 

#### 2.5.3. Video VSDs

Once the six min compilation videos had been created, the Scene and Heard Pro^8^^TM^ application was used to create video VSDs with hotspots. Each six min video was uploaded into the Scene and Heard Pro^TM^ app and programmed with 12 VSDs, with a VSD appearing approximately every 30 s. The researcher then programed two or three hotspots per VSD for a total of 25 hotspots per 6 min video. The 25 hotspots in each VSD were either a one-to-two-word label (*n* = 17) or a relevant sound effect (*n* = 8).

The vocabulary selected for programming with a hotspot, and the label used, was based on the featured activity, animal, or YouTube channel video, and was designed to support the communication goals of each child. Vocabulary was selected based on concepts reported by parents as motivating for the child, as well as the researcher’s judgment of “age-appropriateness” as informed by participant observations and communication with educational staff. Please refer to Table 2 for an example of the vocabulary programmed into the hotspots. For example, one of Maggie’s communication goals was to increase her use of expanded utterances (i.e., two-word utterances rather than one-word utterances). It was reported that Maggie enjoyed looking at books, so in a video clip of an adult reading to a child, the researcher programmed the hotspot, “Reading book,” to expand upon what might be typically expressed by Maggie as a single word (e.g., “Read”).

A total of 25 hotspots appeared in the 6-min video, with 8 that provided sound effects (in quotation marks above) and 17 that provided a spoken label of the vocabulary item.

**Figure 1:**
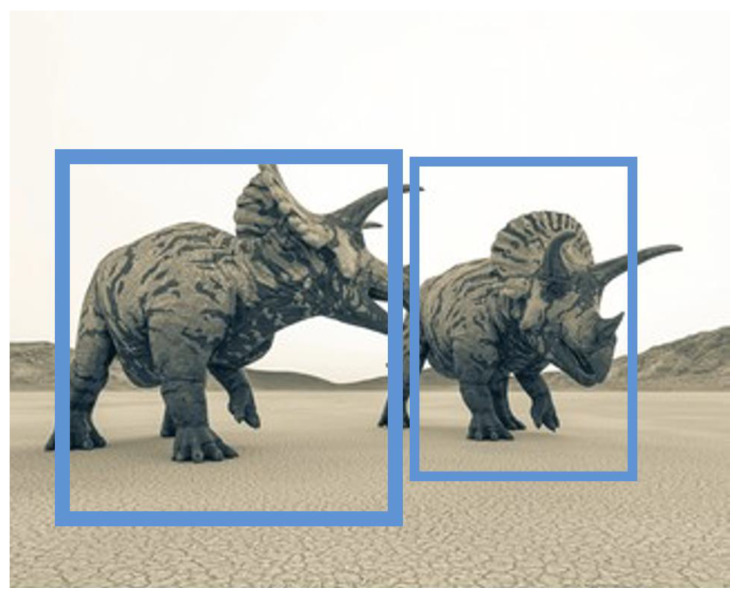
An example of a VSD with hotspots.

Sound effects (e.g., a pig snorting, a truck beeping) were included due to research suggesting that the addition of environmental sounds may be seen as fun by the child and increase participation for participants (Therrien & Light, 2018). During an intervention session, the video played upon activation by the researcher, and when it paused to present the VSD, the programmed hotspots appeared (e.g., a colored outline around the hotspot areas, for example, dinosaurs). Refer to Figure 1 for an example of a VSD with hotspots.

The video in the VSD app shows a clip of dinosaurs walking in the desert. When the video pauses, this still image (VSD) with the hotspots (blue outlines) appears. Upon the activation of a hotspot (e.g., child touches a dinosaur), the recorded output is produced by the app (e.g., “triceratops” for the dinosaur on the left, a “roar!” sound for the dinosaur on the right). The viewer can then choose to activate additional hotspots to communicate more about this clip or press the play button to continue the video.

### 2.6. Experimental Design

The current study utilized a nonconcurrent multiple baseline design (Watson & Workman, 1981; Morin et al., 2024). In a nonconcurrent multiple baseline design, the participants are randomly assigned to a “tier”, that is, the number of baseline sessions that will be conducted prior to the start of intervention data collection sessions (Levin & Kratochwill, 2021). Consistent with single-case research designs, at least five baseline sessions occurred before the intervention began for each participant (Kratochwill et al., 2013). The nonconcurrent multiple baseline design was chosen so that the intervention with participants could begin at different time points to account for common scheduling challenges for participants (Kazdin, 2011). This design has become a regularly used and widely accepted design in AAC intervention research (Boyle et al., 2021; Holyfield et al., 2019; Laubscher et al., 2022a). Nonconcurrent designs may not have the same capabilities as a concurrent design in controlling for certain confounding variables. However, the nonconcurrent design was selected as the most practical and efficient method for this intervention study due to its flexibility and consideration of participant schedules, particularly in a preschool setting to account for absences and pull-out therapies (e.g., speech therapy). 

#### 2.6.1. Independent Variable

The independent variable in this study was an intervention package consisting of video VSDs with hotspots and brief instructional sessions lead by the researcher. The purpose of the instructional sessions was to teach the participants how to use the video VSD and how to activate a hotspot. All the instructional sessions followed explicit instruction procedures (i.e., model, guided practice, independent practice; Archer & Hughes, 2010). Least-to-most prompting (Wolery & Schuster, 1997) was utilized as needed during guided practice to support the participants in activating the hotspots.

Prior to the start of intervention, all the children with NDD participated in two instruction sessions, and all the neurotypical peer participants engaged in one instruction session. Because there were more neurotypical peers than children with NDD, and in order for each child with NDD to participate in two instructional sessions, each peer only needed to appear in one instructional session. These instructional sessions focused on the utilization of video VSDs, including the use of hotspots. The researcher first modeled the use of the video VSD (i.e., activating a hotspot on two VSDs). Then the participants practiced using the video VSD together during structured communication opportunities provided by the researcher with least-to-most prompts provided by the researcher, as necessary. Finally, the participants demonstrated use of the video VSD independently (i.e., activating a hotspot). The participants had three or more independent structured communication opportunities each during these instructional sessions. For more information regarding the video VSD training procedure script, please see Patenaude (2025).

#### 2.6.2. Dependent Variable

The primary dependent variable in this study was the total number of symbolic communicative acts expressed by the participant with NDD per session per min. A symbolic communicative act (S. E. Chapin et al., 2022; Laubscher et al., 2022a; Therrien & Light, 2016, 2018) was defined as the use of spoken words or word approximations, multi-word utterances, activation of hotspots with speech output, touching of images on the screen (no hotspots), manual signs, or conventional gestures (e.g., shaking head no). Communicative acts were counted as a unique occurrence if at least 2 s passed in between two consecutive congruent (i.e., topographically similar) acts (e.g., activation of same hotspot) and were related to the stimuli presented in the video (Laubscher et al., 2022a). Because an aspect of a video VSD intervention package included the video pausing as a cue for the participants of communication opportunities, the duration of interaction sessions varied from baseline to intervention. For the purposes of this paper, we have adopted the most conservative approach of reporting communicative acts per interaction session per min in order to address the variability in the length of the interaction sessions. For more information regarding the symbolic communicative act rules utilized in this article, please see Patenaude (2025). 

For the purpose of this study, symbolic communicative acts were measured both in and out of a participant’s structured turn. A turn was defined as a predetermined time in which the tablet was directed towards the child. Turns occurred for each participant on an alternating basis. Each participant had six turns per session (i.e., each six min video had a total of 12 turns, six for each of the two participants in a 6 min video). The purpose of the turns was to clearly designate the communicative opportunity for a particular child. Each session contained opportunities for both the participants with NDD and their peers to take turns. Though the primary dependent variable in this study was the total number of symbolic communicative acts per session per min, we also provide information on the communicative acts as divided into in-turn and out-of-turn occurrences so that the reader can distinguish between the two types of communication opportunities. 

A participant’s turn began when the researcher pressed the play button on the video at the beginning of their designated turn. The tablet was held in a neutral position, viewable by both participants, and approximately 18 in away from the two children. Then, at the 20 s mark, the tablet was moved within arm’s reach to the target child (approximately 10 in), while still being viewable by the other participant. After moving the screen closer to the participant, the researcher took an expectant 5 s pause (e.g., looked at the participant with raised eyebrows). If a communication act occurred, the researcher provided a conversational recast and/or expansion (Cleave et al., 2015), defined as the researcher repeating some or all of the participant’s utterance and adding additional information on the child’s act (e.g., “Elmo! He’s wearing glasses.”). If the participant did not engage in a communicative act, the researcher cued the child by saying, “I see a [dog]. What do you see?” while touching the screen (S. E. Chapin et al., 2022). A turn would continue if the participant continued to express appropriate communicative acts, with an upper limit of 20 communicative acts per turn. A child’s turn was completed when 5 s passed with no communicative act from the participant. Once a participant’s turn ended, the other participant’s turn began (which also marked the start of measurement of out-of-turn communicative acts for the child whose turn had just ended). It should be noted that regardless of which participant was currently taking their “turn”, all communicative acts exhibited by either child were encouraged and recorded.

### 2.7. Procedures

#### 2.7.1. Setting

All the sessions took place in a small private room at the preschool and used a child-sized table with two child-sized chairs. The researcher sat on the floor approximately one foot in front of the table at which the participants were seated. 

For all sessions, the participant with NDD was paired with a neurotypical peer. The procedure to select a peer followed three steps: (1) identify the consented peers in attendance; (2) if more than one peer was available, remove the most recent partner from consideration for the candidate pool; (3) select a peer at random from the remaining candidate pool. Due to varying availability, neurotypical peers participated in between 6 and 10 total sessions.

The peer selected for each dyadic interaction was chosen in this way for three reasons. First, a rotating set of neurotypical peer participants may promote the generalization of developed skills across peer communication partners (Thiemann-Bourque, 2012). Second, the use of multiple peer participants also more closely mirrors a typical educational setting, in which children have opportunities to interact with multiple partners (Chen et al., 2019; Lin et al., 2019). Finally, having multiple available neurotypical peers participate addressed potential scheduling complications (e.g., absent participants). 

#### 2.7.2. Baseline

Baseline sessions began with both participants sitting together at a table with the researcher. The two children were seated side by side and the researcher was seated across the table from them. The researcher told the participants that they would watch a video and talk about it together. The researcher then pressed the play button on the tablet device and played one of the compilation videos (specific to the child with NDD in the dyad). The same compilation videos were used in both baseline and intervention sessions; however in baseline no hotspots were available on the tablet device. 

Approximately every 30 s, the researcher provided a turn for one of the two children on an alternating basis. There were an equal number of turns for each participant in each baseline and intervention session (i.e., 12). In baseline, there were no VSDs and therefore, the videos did not pause. During each participant’s turn in baseline, at approximately 20 s, the researcher provided a verbal cue if the target participant had not expressed a communicative act (i.e., “I see a ___. What do you see?”). When a participant’s turn was completed, the tablet returned back to its initial position (i.e., in front of the researcher). The next child’s turn then began, and the alternating turns continued via the same procedure. The participants participated in between 5 and 9 baseline sessions based on the randomly assigned tiered baseline procedures utilized in nonconcurrent multiple baseline designs (Levin & Kratochwill, 2021).

#### 2.7.3. Intervention Procedures

**Figure 2:**
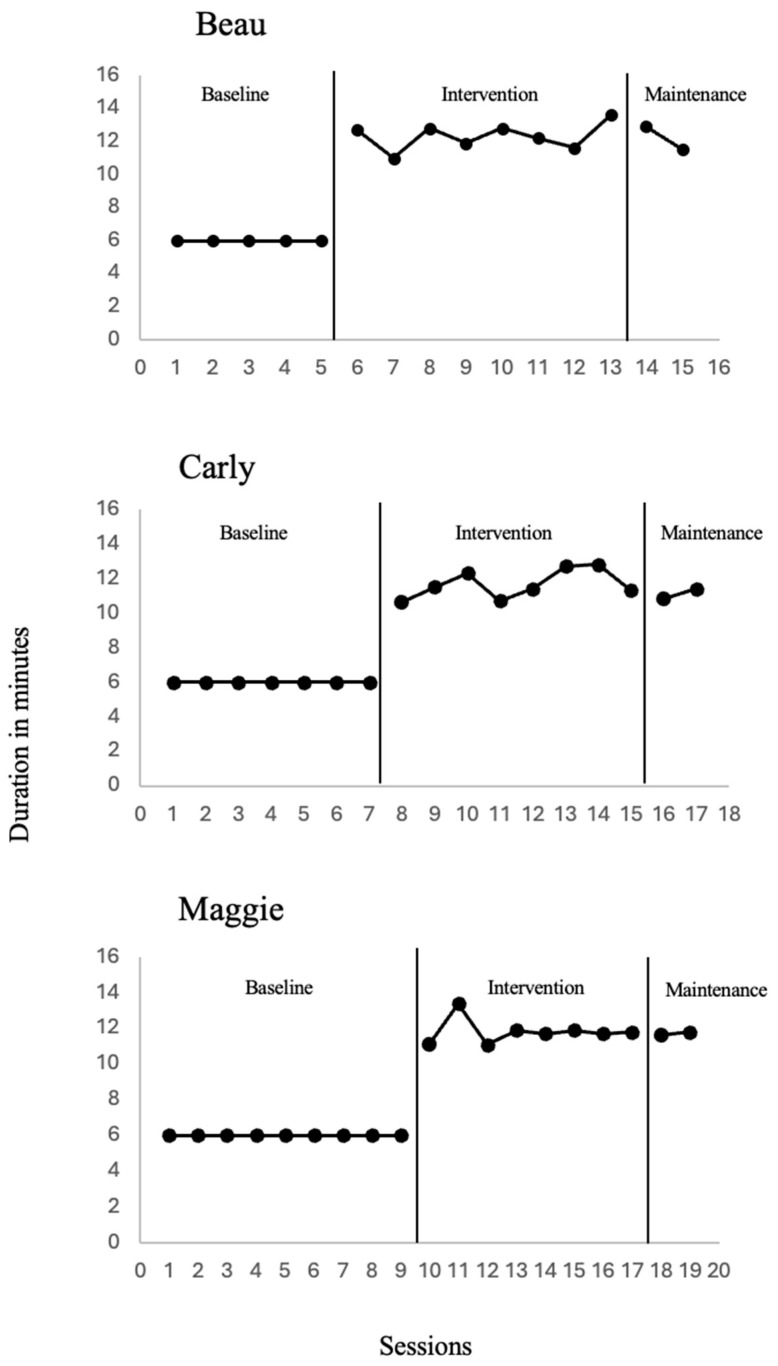
Duration of interaction sessions in minutes.

Intervention sessions were structured the same as the baseline sessions, except the video shown was in a video VSD format with programmed hotspots. The child with NDD took the first turn in each trial. At approximately the 20 s mark, the video paused, and two or three hotspots (as indicated by solid blue boxes) on the VSD appeared. At this time, the researcher indicated which child’s turn it was by moving the tablet closer to them, while still in view of both participants. The researcher gave an expectant 5 s pause. If no communicative act was performed by the target child, the researcher cued by saying, “I see a ___. What do you see?” while touching a hotspot. If a communicative act occurred after the cue, the researcher provided a conversational recall and/or expansion on the child’s act. A child’s turn was completed when 5 s passed with no symbolic communicative act from the participant whose turn it was, or a maximum number of communicative acts was exhibited (i.e., 20). At that point, the researcher continued the dyadic interaction by saying, “Okay, let’s keep going,” while moving the tablet back to the original neutral position in front of the researcher and pressing the play button. At this time, the next participant took their turn following the same procedure. All the communication acts exhibited by either participant, whether in or out of their turn, were encouraged and recorded. The intervention sessions were approximately 10–12 min in duration for each of the 6 min videos. Refer to Figure 2 for the duration of the interaction sessions in minutes.

### 2.8. Data Analysis

The data for symbolic communicative acts were graphed, and the graphs were analyzed using visual analysis to examine the level, trend, variability, overlap, and immediacy of effect (Kratochwill et al., 2013). Tau-U was calculated using an online calculator to determine the effect sizes. According to Vannest and Ninci (2015), effect sizes below 0.2 indicate a small effect size, 0.2–0.6 a moderate effect size, 0.6–0.8 a large effect size, and above 0.8 a very large effect size.

### 2.9. Coding

All baseline, intervention, and maintenance/follow-up sessions were video-recorded for coding. The primary researcher in this study was a graduate level student in a Special Education Ph.D. program. She used the definitions (e.g., symbolic communicative act) created to collect and code all the data. The primary researcher both collected and coded all the data; therefore, she was not masked to the treatment condition. 

### 2.10. Interobserver Agreement and Procedural Integrity

Interobserver agreement (IOA) was completed by a second graduate student in a Special Education program, masked to the treatment condition, and who coded 20% of the baseline, intervention, and maintenance/follow-up sessions selected at random for each participant with NDD. The second coder was trained using videos not used for session coding until agreement of 80% or higher was obtained on three consecutive videos. To calculate IOA, both the researcher and the second coder recorded all words, word approximations, and multi-word utterances heard during the 9 sessions that were coded for IOA. A data sheet was created by the researcher to record all the symbolic communicative acts of the participants. Point-by-point agreement of communicative acts (including, as appropriate, spoken vocabulary or hotspot activations with speech output) in each of the participant’s six turns per video was then calculated. IOA was calculated by dividing the number of agreements by the number of agreements plus disagreements and multiplying that by 100. For example, in turn 1, if the coder heard two spoken words (i.e., dog, puppy), and the researcher heard one spoken word (i.e., puppy), they would have an IOA of 50% for spoken words. IOA averaged 91% across all phases of the study, including baseline (range = 88–100%; average = 94%), intervention (range = 81–97.5%; average = 87%), and maintenance/follow-up (range = 87.5–93%; average = 90%) for both in- and out-of-turn communicative acts. The most common disagreements in the calculation of IOA between the researcher and coder were caused by differences in interpretations of speech approximations of participants (e.g., one coder coded the occurrence of a single word, while the other recorded the occurrence of two words). 

For all the baseline, intervention, and maintenance/follow-up sessions, the researcher made use of a script. Procedural integrity was measured on 20% of the baseline, intervention, and maintenance sessions videos by the second graduate student coder and was calculated by dividing the number of steps completed correctly by the total number of steps and multiplying by 100. Procedural integrity averaged 99% across all phases of the study (range = 94–100%).

### 2.11. Social Validity

Social validity was completed by the participants with NDD, the neurotypical peer participants, and the teachers of the participating students. All the child participants were shown pictures of themselves engaging in the research activity and were asked their opinions on the activity with the researcher and peers, watching the videos without hotspots, and watching the videos with hotspots. All the child participants sorted the pictures of themselves engaging in the various aspects of the activity and were asked to use a Talking Mats approach to sort the pictures into “LIKE” or “DON’T LIKE” categories. Talking Mats are a communication support tool often used with adults with dementia or intellectual disabilities to support individuals in conversating by sorting images into various categories (Stans et al., 2019).

The teacher participants were shown two 2 min video clips of each student with NDD, one clip of the child in baseline and one clip of the child in intervention phases selected at random. They were masked to which phase they were watching. The teachers were then asked to complete a series of Likert scale and open-ended questions regarding in which video they felt the student was communicating more effectively and their opinions on the intervention.

## 3. Results

**Figure 3:**
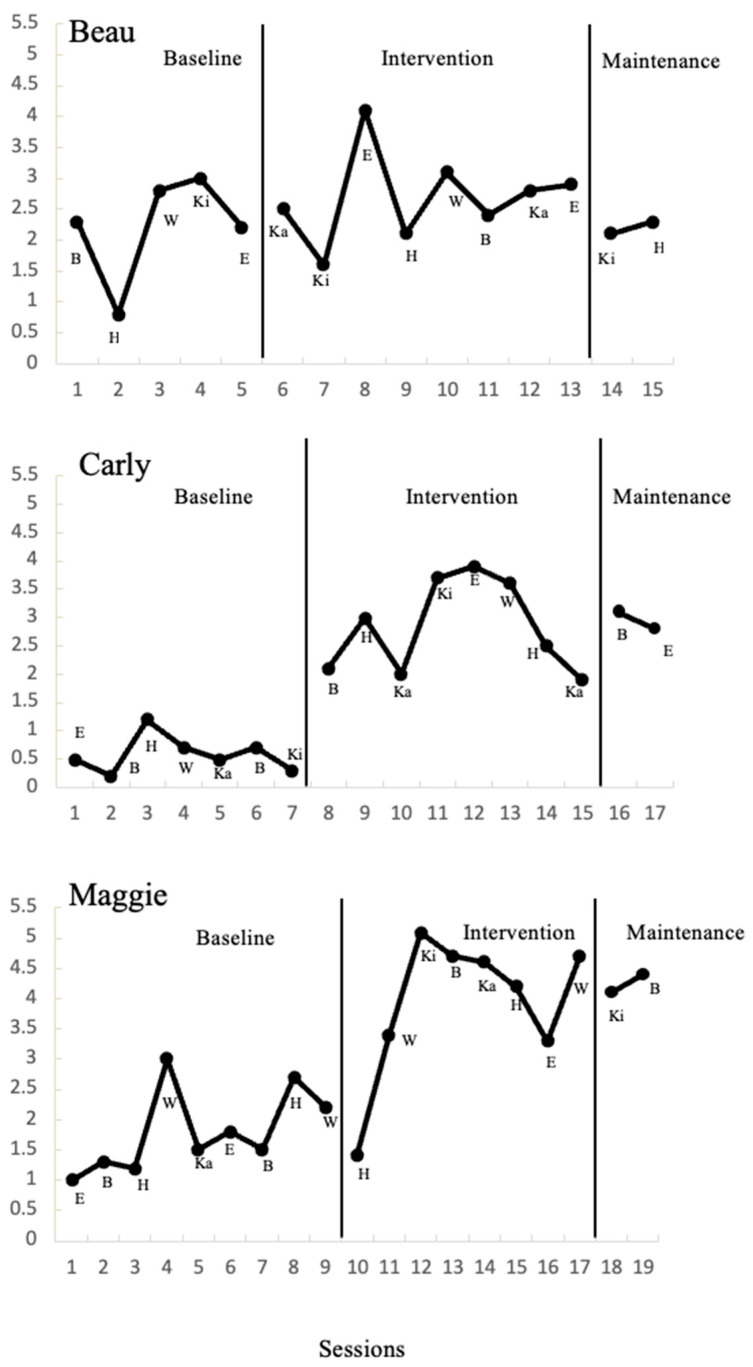
Frequency of symbolic communication acts in- and out-of-turn per minute taken by participants with NDD. Note. Neurotypical peer partner indicated by initials for each session; B (Bethany); E (Ellie); H (Harry); W (William); Ki (Kim); Ka (Kate).

**Figure 4:**
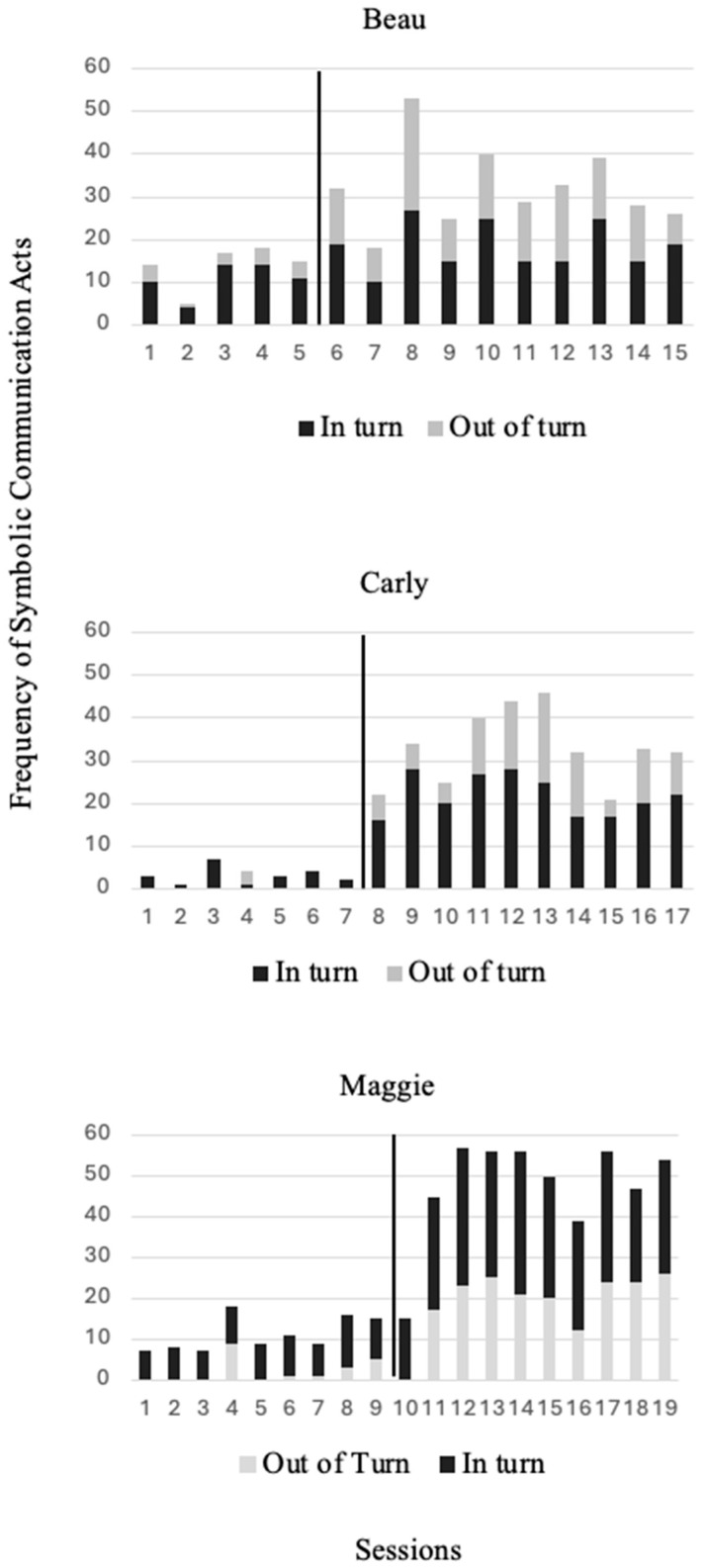
Frequency of in- and out-of-turn symbolic communication acts of participants with NDD per session.

All three participants with NDD demonstrated an increase in the number of symbolic communicative acts following the introduction of the video VSD intervention package (Tau-U of 0.28–1.00 across all participants). Please refer to Figure 3 for the frequency of symbolic communication acts taken in- and out-of-turn per min per session and Figure 4 for the total frequency of symbolic communicative acts in- and out-of-turn per session. In baseline, the total frequency of communicative acts per session ranged from one to 18. During intervention sessions (i.e., when the video VSD app was available), the participants demonstrated an increase in the frequency of communicative acts per session, with a total range of 15–57 communicative acts. 

All the participants participated in 5–9 baseline sessions, eight intervention sessions, and two maintenance/follow-up sessions. 

The data for this study is presented in two ways—total frequency of symbolic communicative acts per session, and frequency of symbolic communicative acts per session per min (i.e., total communicative acts per session divided by the length of the session, in mins)—to account for the change in session duration for all the participants from baseline to intervention. With respect to the total frequency of communicative acts per session, for two of the three participants, the frequency of communicative acts in all intervention and maintenance/follow-up sessions were equal to or higher than the highest data point observed in baseline. For one participant, seven of the eight intervention data points, as well as both maintenance data points, were higher than the highest baseline data point. Furthermore, for all three participants with NDD, there was evidence of generalization across communication partners, as the impact of the video VSD intervention was observed regardless of the neurotypical peer who participated in the dyad. 

With respect to symbolic communicative acts per session per min, for one participant, all intervention and maintenance/follow-up data points were higher than the highest baseline data point (Carly). For Maggie, seven of the eight intervention data points as well as both maintenance/follow-up data points were higher than the highest baseline data point. Beau exhibited more variability in his communicative acts per session per min, with four intervention data points being higher than or equal to his highest baseline data point and four being lower than his highest data point. Detailed information on participant performance is provided below.

### 3.1. Beau

Beau participated in five baseline sessions, eight intervention sessions, and two maintenance/follow-up sessions over approximately 105 days. Beau’s baseline data averaged 13.4 total symbolic communicative acts (range = 5–18) per session. Beau demonstrated an increase in performance immediately upon the introduction of the video VSD application. Following the introduction of the video VSD intervention, the total frequency of communicative acts per session was higher than the highest point observed in baseline in all but one session (in which the total number of communicative acts was equal to the highest in baseline) and averaged 33.6 symbolic communicative acts per session (range = 18–53). Beau’s performance in maintenance/follow-up sessions averaged 27 symbolic communicative acts (range = 26–28), both sessions being within the range of his intervention data points, and neither below the highest data point in baseline sessions.

The duration of Beau’s interaction sessions increased with the introduction of the video VSD intervention. In all baseline sessions, the total duration of each session was 6 min long, as the video did not pause to cue a communication opportunity. The total symbolic communicative acts per session per min for Beau averaged 2.2 symbolic communicative acts in baseline (range = 0.8–2.8). During the intervention sessions, the duration of Beau’s interaction sessions increased to an average of 12.3 min (range = 11–13.6 min). In intervention, the total symbolic communicative acts per session per min for Beau increased to an average of 2.7 symbolic communicative acts (range = 1.6–4.1). The calculated Tau-U effect size measure for Beau’s communicative acts per session per min was 0.28, indicating a moderate effect size (Vannest & Ninci, 2015). Refer to Figure 3 for the frequency of symbolic communicative acts per min per session.

### 3.2. Maggie

Maggie participated in nine baseline sessions, eight intervention sessions, and two maintenance/follow-up sessions over approximately 105 days. Maggie’s total frequency of symbolic communicative acts in baseline sessions averaged a total of 10.8 symbolic communicative acts per interaction session (range = 6–18). Maggie engaged in more total symbolic communicative acts within the video VSD activity, as opposed to in baseline (i.e., without the video VSD), in all but one session. In Maggie’s first intervention session, she engaged in 15 total symbolic communicative acts, which was below her highest baseline session (i.e., 18). During the intervention sessions, Maggie produced an average of 46.5 total symbolic communicative acts (range = 15–57) per interaction session. Maggie’s total symbolic communicative acts in maintenance/follow-up sessions averaged 49.5 symbolic communicative acts (range = 47–52), both sessions being within the range of her intervention data points and neither below the highest data point in baseline sessions.

The duration of Maggie’s interaction sessions increased with the introduction of the video VSD intervention. In all baseline sessions, the total duration of each session was 6 min long. The total symbolic communicative acts per session per min for Maggie averaged 2.0 symbolic communicative acts in baseline (range = 1–3). During intervention sessions, the duration of Maggie’s interaction sessions increased to an average of 11.8 min (range = 11.1–13.4 min). In intervention, the total symbolic communicative acts per session per min for Maggie increased to an average of 3.9 symbolic communicative acts (range = 1.4–5.1). Maggie demonstrated an increase in symbolic communicative acts per session per min from baseline to intervention with a Tau-U of 0.83, a very large effect size (Vannest & Ninci, 2015). Refer to Figure 3 for the total frequency of symbolic communicative acts per min per session.

### 3.3. Carly

Carly participated in seven baseline sessions, eight intervention sessions, and two maintenance/follow-up sessions over approximately 60 days. Carly engaged in more total symbolic communicative acts within the video VSD activity than in the baseline condition. Her baseline data averaged 3.4 total symbolic communicative acts (range = 1–7) per session. During intervention sessions, Carly’s data averaged 33 total symbolic communicative acts (range = 22–46). There was no overlap between Carly’s intervention data points and baseline data points. Carly’s performance in maintenance/follow-up sessions averaged 32.5 total symbolic communicative acts (range = 32–33), both sessions being within the range of her intervention data points and neither below the lowest data point in baseline sessions.

The duration of Carly’s interaction sessions increased with the introduction of the video VSD intervention. In all baseline sessions, the total duration of each session was 6 min long. The total symbolic communicative acts per session per min for Carly averaged 0.6 symbolic communicative acts in baseline (range = 1–3 min). During intervention sessions, the duration of Carly’s interaction sessions increased to an average of 11.7 min (range = 10.6–12.8 min). In intervention, the total symbolic communicative acts per session per min for Carly increased to an average of 2.8 symbolic communicative acts (range = 1.9–3.9). Carly exhibited an increase in symbolic communicative acts per session per min with no overlap between baseline and intervention data points, demonstrating a Tau-U effect size of 1.0. Refer to Figure 3 for the total frequency of symbolic communicative acts per min per session.

### 3.4. Communication Modalities

In addition to the frequency of communication acts, the modalities of the communication acts were evaluated. The communication modalities that were measured included speech (single-word and multi-word utterances), conventional signs/gestures, touching the tablet screen (no hotspots), activation of label hotspots, and activation of sound hotspots. Refer to Table 3 for the average number of communication acts per modality.

During intervention, Beau’s communication increased in frequency of in-turn communicative acts across all modalities except touching the tablet screen (no hotspots), increasing from 8.4 communication acts in baseline to 19.5 in intervention on average. More specifically, Beau demonstrated an increase of 0.4 symbolic communicative acts per min in the intervention condition, including increases in both single-word and multi-word utterances. When evaluating speech acts combined (i.e., single- and multi-word utterances), Beau increased speech production by 0.7 communication acts on average. Carly’s communication increased in frequency of in-turn communication acts across all modalities (including speech acts combined), increasing from three communication acts in baseline to 22.2 in intervention on average. Carly’s speech acts combined increased 0.3 communication acts on average during the intervention phase. Carly’s total communication acts per min, including single-word and multi-word utterances, increased by 2.2 symbolic communicative acts on average from baseline to intervention. Maggie’s communication increased in frequency of in-turn communication acts across all modalities (including speech acts combined) except conventional signs/gestures from nine communication acts in baseline to 29.5 in intervention on average. Maggie’s total communication acts per min, including single-word and multi-word utterances, increased by 2.1 symbolic communicative acts on average. All the participants experienced an increase in combined single- and multi-word speech in the intervention condition in this study.

### 3.5. Social Validity

For each of the three participants with NDD, a classroom teacher was asked to complete social validity measures. A total of two teachers completed the social validity questionnaire, as one teacher had two participants in her classroom. The teacher with two participants in her classroom answered the social validity questions separately for each participant. All the child participants completed a Talking Mat activity to contribute their perspective on the research activities.

#### 3.5.1. Teachers

Mrs. Blick completed social validation measures for two participants with NDD (Maggie and Carly), and Ms. Morgan completed the measures for one participant with NDD (Beau). Please refer to Patenaude (2025) for the teacher and child participant social validity results. After watching two 2 min video clips, one of a child’s intervention session and one of a baseline session (with conditions masked), both teachers reported that the participants communicated more effectively in the intervention video clips. In response to Likert scale questions, the teachers reported that the intervention was usable in an early childhood setting by an early childhood educator (*n* = 3, strongly agree) and believed the activity would be beneficial for all students, with (*n* = 3, strongly agree) or without NDD (*n* = 3, strongly agree). Both teachers also noted that the children enjoyed participating in the research study (*n* = 3, strongly agree), that the activity supported peer communication (*n* = 3, strongly agree), and that it was appropriate for their students (*n* = 3, strongly agree). Mixed results were obtained in response to the question, “The child’s communication skills improved as a result of the intervention”, including “strongly agree”, “agree”, and “neither agree nor disagree”. It is unclear if the teachers interpreted this question as pertaining to generalized improvement of communication skills outside of the intervention activity, or whether their comments were focused on the child’s performance while they had access to the video VSD.

In response to open-ended questions, the teachers reported that the participants experienced improved social interactions, and two participants demonstrated increased communication as a result of the study. Mrs. Blick saw an increase in communication and peer interactions for Maggie after sessions and noted that she seemed more “revved up” for social interactions with peers after sessions. Both teachers reported that they would be interested in using this intervention in their classrooms with their students to increase turn-taking, spontaneous language, engagement, and communication. Both teachers said that they would recommend this intervention to others.

#### 3.5.2. Child Participants

All nine child participants completed an adapted Talking Mat approach to answer social validity questions regarding the research study activities (Stans et al., 2019). In a Talking Mat approach, individuals sort pictures into “LIKE” and “DON’T LIKE” categories to express their preferences. The participants were shown three screenshots taken from the video recordings of research activities including one of the participant and a peer with their faces visible, while facing the researcher; one with themselves and a peer watching a video without hotspots (e.g., dinosaur video); and one of themselves and a peer watching a video with hotspots visible in the screenshot (e.g., dinosaur video paused on VSD with hotspots visible). Additionally, all the participants were shown 2–3 pictures of things reported to be disliked by themselves and parents during the research study. 

Based on the responses obtained via the Talking Mats procedure, all the research activities were reported to be enjoyed by seven of the nine participants. Two participants (Beau and Carly) reported they disliked the activities with peers and researchers; however, Beau reported enjoying watching videos with peers and watching videos with hotspots. Additionally, Carly reported disliking all the pictures shown to her during the social validation activity. It should be noted that the adapted Talking Mats approach may not have accurately captured the preferences of these young children. Talking Mats have been utilized widely with adolescents and young adults as communication supports (Murphy & Oliver, 2013; Darvell & Bradshaw, 2023); however, future research is needed to evaluate their efficacy with young children. Anecdotally, in contrast to the negative responses they provided in the Talking Mats activity, both Beau and Carly provided assent and left their preschool classroom quickly when asked if they would like to engage in the research activities, and demonstrated strong engagement in the activity when engaging in it (Patenaude, 2025). It should, however, be noted that they sorted some or all of the images into the “DON’T LIKE” category during the social validity activity. The social validity results of the child participants should be interpreted cautiously.

## 4. Discussion

This study supports and extends prior findings on the positive impacts of a video VSD approach on communication for children who use or could benefit from the use of AAC. Past research has demonstrated increases in communication for children with NDD and speech or language delays ranging in age from preschoolers to young adults (Babb et al., 2021; Caron et al., 2018; S. E. Chapin et al., 2022). The current study provides evidence that video VSDs with brief instructional sessions can be used in peer interactions with preschool-aged children with NDD and lead to similar results, with moderate to very high increases in the number of symbolic communicative acts for all three participants (Tau-U from 0.28 to 1.00). These results are of particular interest due to the increase in communication exhibited by all three participants with NDD while interacting with all six neurotypical peers. Children with disabilities have been found to have smaller peer networks (Kasari et al., 2011), and activities which can quickly and easily support peer interaction are of particular interest.

This study demonstrates that video VSDs and brief instructional sessions can be an effective communication support during video viewing, a highly preferred activity for preschool children with and without NDD. One or more of the following unique qualities of video VSD technology may have contributed to the positive results (Caron et al., 2018; Light et al., 2019).

### 4.1. Communication Support

Traditional AAC systems can be challenging for young children to use due to decontextualized images (e.g., line drawings) and the need to triangulate attention (J. W. McCarthy et al., 2018; Worah et al., 2015). Additionally, much AAC research with children focuses on requesting (Iacono et al., 2016; Light & McNaughton, 2015). VSDs and video VSDs offer an alternative to traditional systems that address these common challenges. In the current study, the video VSD intervention package was found to be an effective communication support for preschool-aged children with NDD. All the participants demonstrated an increase in the frequency of symbolic communicative acts with no negative impact on speech with the use of a video VSD approach during a shared video activity with peers. Much of the increased change in communication can be contributed to the use of hotspots contained in the VSD (refer to Table 3). However, all the participants increased their combined single- and multi-word utterances from baseline to intervention phases on average as well. In the video VSD approach, the participants did not have to triangulate their attention between the communication device, their communication partner, and the topic being discussed, due to the vocabulary being programmed directly into the device playing the videos. With vocabulary programmed directly into the video, the participants were able to learn to utilize the video VSD approach to support communication during the activity. 

As in previous studies using video VSD technology, communication “cues” were naturally embedded directly into the video VSD as well. In this study, the appearance of the VSDs with hotspots when the video paused provided a natural cue for children of a communicative opportunity, as previous research has demonstrated (Babb et al., 2021; S. E. Chapin et al., 2022). 

Due to the nature of the video VSD intervention package, interaction session durations in the intervention and maintenance/follow-up sessions were longer than the duration of the interaction sessions in baseline. It is possible that some of the increases in participant communicative behaviors were in part due to the longer interaction session durations. It should be noted that an integral aspect of video VSDs is that the video programmed into the application pauses and VSDs appear with hotspots present that produce speech output (Caron et al., 2018; Light et al., 2019). For the purposes of this paper, the researchers utilized the most conservative approach of reporting symbolic communicative acts per interaction session per min in order to remove the variable of the increase in duration of interaction sessions. However, it must be noted that it is possible some of the increases in the frequency of communicative acts could be attributed to the increased durations of intervention sessions. It is of interest to consider, however, whether the increase in duration is in and of itself a positive thing, as this means that there was an increased amount of time and opportunities in which these peers are interacting in a language-based activity. Therefore, longer interaction durations provide more opportunities for peer modeling and language development. The relatively short duration of this study may not have captured the increases that may have been observed as a result of the increase in duration of interaction sessions across an extended period of time.

The impact of the increased interaction session durations was most notably seen in Beau’s symbolic communicative acts per min per session. The total frequency of Beau’s symbolic communicative acts increased from baseline to intervention, with an average increase in in-turn symbolic communicative acts of 18.9 and 14.8 out-of-turn symbolic communicative acts during the intervention sessions. Though Beau had an increase in total symbolic communicative acts, when evaluating his total symbolic communicative acts per session per min, his effect was moderate (Tau-U = 0.28); thus, Beau’s results should be interpreted with caution. It is possible that the increases seen in Beau’s symbolic communication were in part due to the increased duration of sessions, which provided more opportunity for communication acts to occur. It is of interest to note, however, that Beau engaged in a substantially higher number of out-of-turn symbolic communicative acts in the intervention sessions. During baseline, Beau was largely silent when it was the turn of his peer. During intervention sessions, as peers made use of the video VSD, they were providing natural language models for Beau. Anecdotally, Beau was observed during the turns of his peers to be observing his peer’s communicative behaviors and was also observed to reach over the peer to activate hotspots during their turns. Therefore, it is possible that for Beau, the increased duration of the interaction session provided more opportunities for peers to model various forms of communicative behaviors, resulting in more symbolic communicative acts (including hotspot activations and imitations of speech) by Beau.

In contrast to past research on peer-mediated interventions (Chan et al., 2009; Chang & Locke, 2016; Therrien & Light, 2016, 2018), no extended formal training was required in this study to instruct neurotypical peers on how to interact with children with NDD to see increases in their communication. In the current study, all the participants participated in two (children with NDD) or one (neurotypical peers) brief instructional sessions. The sessions served as an introduction to video VSDs (e.g., activation of hotspots), in which the researcher first provided a model, followed by guided practice and independent practice. Although the researcher did provide models, it was anecdotally observed that all the participants began independently activating hotspots after only 1–2 models. As in both S. E. Chapin et al. (2022) and Babb et al. (2021), the current study provides evidence that the introduction of a video VSD activity typically results in a sharp increase in communication, and only very limited instruction is required.

### 4.2. Child Interests

Incorporating children’s interests into instructional materials can significantly enhance their motivation and engagement (Fedewa et al., 2024). Utilizing content that reflects these interests also provides a natural context for teaching meaningful vocabulary. Given the increased engagement seen with materials that incorporate child interests, activities that reflect the preferences of children with and without NDD may promote more positive social interactions.

Research by Babb et al. (2021) with autistic adolescents demonstrated that incorporating the interests of both individuals with disabilities and their neurotypical peers can increase peer engagement and social interaction. In the current study with preschool children with NDD, the researchers similarly integrated the interests of all the participants into a video VSD activity. This approach included both preferred content (i.e., video clips) and a preferred activity (i.e., video watching), resulting in consistently high engagement across the participants. 

It is of interest to note that both the children with NDD and children without NDD demonstrated strong engagement even when viewing clips that reflected their peers’ interests and preferences rather than their own. This introduces another potential benefit of a video VSD approach: the provision of opportunities for children to learn about the interests of a peer, and even to communicate with one another about non-preferred content. For example, worms were an animal that were reported to be non-preferred for Beau by his parent. While watching video clips of worms, however, Beau was attentive to the videos, and positively engaged both in spoken communication (i.e., single-word approximations) and the activations of label hotspots (i.e., “worms” and “dirt”). In this way, the activity not only supported communication (e.g., increase in symbolic communicative acts), but it also may have fostered an understanding of the interests and preferences of classmates.

Furthermore, engaging with peers around shared and diverse interests may help children expand their expressive vocabulary beyond their own preferred topics. By embedding communicative opportunities into a typically solitary activity, the video VSD intervention transformed video watching into an interactive experience for all the participants (Fridberg et al., 2021; Kucker et al., 2024). This method presents a promising approach to enhancing peer communication and social engagement among children with and without NDD.

### 4.3. Interaction Support

Traditionally, peer support interventions aimed at increasing communication between children with and without NDD require extensive training to prepare neurotypical peers to effectively support their peers with disabilities in communicating (Chang & Locke, 2016). In this intervention, all neurotypical peers learned how to make use of the video VSD in a single instructional session, typically after one or two models of hotspot activation. They also reported positive perceptions of the activity. As also seen in previous VSD research (Boyle et al., 2017; Laubscher et al., 2019; Therrien & Light, 2016, 2018), social validity measures indicated that all six neurotypical participants reported enjoying engaging in the activities with peers and the researcher, watching videos with friends, and watching videos and using the buttons (i.e., hotspots) to talk about the videos. This provides additional evidence that a video VSD intervention is both easily implemented and positively received by participants. 

In a small number of previous studies, there was minimal participation by adults. For example, in studies by Therrien and Light (2016, 2018), the researcher did not hold the tablet device or allocate turns, as in the current study. In the current study, the researcher maintained control of the tablet throughout the session to address the potential challenge of one child in the dyad seeking to maintain exclusive control of the tablet, as has been previously observed in past research (Therrien & Light, 2016). 

There are benefits to identifying strategies to reduce or eliminate the involvement of the researcher during the video VSD activity. For example, a more natural interaction may occur without an adult researcher present. In this study, the researcher kept her speech to a minimum by communicating only to expand upon communicative acts by the participants, to offer an equal number of turns to each participant, and to encourage and record communication acts regardless of whose structured turn it was. However, the extent of the impact researcher involvement had on the results cannot be known for certain. Future research should investigate the impact of strategies to reduce (or even eliminate) researcher participation in video VSD interventions, while ensuring equal communication opportunities for children with and without NDD.

### 4.4. Clinical Implications

The video VSD intervention package demonstrated numerous benefits for the participants with NDD, including interaction opportunities with multiple neurotypical peer communication partners, the introduction of the use of a communication support, and exposure to naturalistic peer models of communication. None of the children with NDD currently use an AAC device, though all three experienced an increase in communication with the introduction of the video VSD intervention package. In addition, their neurotypical peers were exposed to technology-based communication supports, which many may not have encountered before. 

As a social validity measure, the teachers of the participants evaluated child communication with and without the use of the video VSD approach. All the participating teachers reported their students with NDD communicated more frequently and effectively when using the video VSD as opposed to with no communication support (i.e., baseline condition). This study may have contributed to a wider acceptance of video VSD and other forms of AAC within an inclusive preschool setting for children with and without NDD and their teachers. 

Finally, neurotypical peers’ successful usage of this communication support, which for some children functions as AAC technology, served as a model for their peers with NDD. Modeling of AAC use is critical in the learning process for beginning communicators (Beukelman & Light, 2020). Opportunities for all peers to serve as models for one another were naturally embedded into this intervention, creating a unique mutually beneficial and naturalistic experience for preschool-aged children to learn from each other. 

### 4.5. Limitations and Future Research

Despite the promising findings of this study, several limitations should be acknowledged. Although all three participants with NDD demonstrated increases in communication, with effect sizes ranging from moderate to very large, these results should not be generalized to all children with NDD. Additionally, due to the small sample size (*n* = 3), the results cannot be generalized to the larger population. Future research should conduct a similar investigation with a larger sample size to replicate findings and further promote generalization of the findings. 

Another limitation in this study is that the increases in symbolic communication seen may not be able to be solely attributed to the video VSDs, due to the video VSDs being part of a packaged intervention along with the brief instructional sessions and researcher support. It is unknown to what degree the brief instructional sessions and researcher support contributed to the increases in communication exhibited by the participants. Future research should evaluate the use of video VSDs with minimal additional supports on the frequencies of symbolic communication.

As part of a video VSD intervention, videos naturally pause to cue a communication opportunity, contributing to the differences in session durations in baseline and intervention. Despite the authors choosing to take the most conservative approach and reporting the frequency of symbolic communication acts per session per min, it is possible that the increased duration, allowing for more opportunities for communication between participants, in part contributed to increases in the frequency of symbolic communication acts. It should also be noted that though not formally analyzed, no indication of communication fatigue was evaluated. As part of the assent procedures, the authors both obtained assent from the participants before each session and paid close attention to signs of withdrawal of assent throughout the sessions. 

Additionally, the use of the adapted Talking Mats approach to social validation may not have fully captured the participants’ true perspectives on the study activities. This approach was utilized for this study due to the absence of a speech response requirement, or the ability to read the social validity questions. This approach has not been widely studied with children thus far, and this may have been reflected in child participant responding. The child participant social validation data should therefore be interpreted cautiously. Future research should explore the use of an adapted Talking Mats approach with young children with and without NDD. 

The researcher undertook multiple steps to develop the videos used in the intervention, including consulting with parents regarding their child’s preferences and creating individualized compilation tapes. However, given educators’ extensive responsibilities and limited resources, it may be impractical to expect them to produce customized videos tailored to the diverse interests of each of their students. As previously mentioned, the participants communicated with peers across video clip content, including those of content reported by parents to be non-preferred. This indicates that the participants communicated regardless of having a strong preference for the video clip content. These findings suggest that creating individualized videos may not be necessary to increase social communication between children with and without NDD, though the interests of participants should still be incorporated for engagement and motivation. The methodological approach taken by the authors promoted the incorporation of materials of interest to the participants, to encourage peer social interactions focused on preferred (or non-preferred) activities or items. Though high levels of engagement were observed across all videos, future research should investigate more efficient methods for identifying appropriate video content for use in video-based VSD activities. One potential method could include using highly preferred full videos instead of creating shorter video compilations. Readers should refer to Bhana et al. (2020) for feasible creation of VSD materials.

Another limitation involves the lack of masking during the coding process. The researcher was not able to be masked to the condition of the videos being analyzed, introducing the possibility of bias due to her awareness of the video phase. In addition, although an analysis of changes in vocabulary knowledge and use as a result of the intervention may be of interest in future research, it was determined to be beyond the scope of this study. Finally, future research should examine the broader application of similar interventions and include the formal collection of data from neurotypical peer participants.

## 5. Conclusions

The results of this study show promising evidence that video VSDs along with brief instructional sessions and researcher support can be used to increase the frequency of symbolic communication of children with NDD during an inclusive video activity with their neurotypical peers. Communication interventions, including those that incorporate AAC technology, must go beyond requesting and should support peer social interaction. Incorporating the interests of all children increases engagement and therefore may increase peer interactions during highly preferred shared activities. The findings of the current study highlight the importance of designing AAC interventions that are both meaningful and contextually engaging, advancing inclusive practices that support communication and connection for all children.

## Figures and Tables

**Table 1:** Demographic information for participants.

Name	Age (Gender)	Race/Ethnicity ^a^	Disability	MBCDI ^b^	PLS-5 (Not Passed)
Maggie	4;8 (F)	White	NDD	369/363	L; A; C; F
Beau	4;3 (M)	Multi-racial	NDD	331/206	L; C; S; F
Carly	5;2 (F)	White	ASD	386/207	L; A; C; S; F
Dominic ^c^	4;9 (M)	White	NDD	396/396	-
Bethany	3;1 (F)	White	-	-	-
Ellie	3;2 (F)	White	-	-	-
Harry	4;0 (M)	White	-	-	-
William	4;8 (M)	White	-	-	-
Kate	4;2 (F)	White	-	-	-
Kim	4;2 (F)	White	-	-	-

^a^ Identity markers as reported by parents; ^b^ parents of participants without NDD did not complete the MBCDI; ^c^ Participant was withdrawn from study post baseline data collection, see text for rationale.

**Table 2:** Example of hotspots programmed for a video.

Time	Programmed Hotspots
0:30	Peppa Pig, “splash”
1:00	eating, baby doll
1:30	beachballs, “laugh”
2:00	butterfly, kinetic sand
2:30	Bingo, Bluey, “beep”
3:00	“slurp”, anteater
3:30	triceratops, “roar”
4:00	“neigh”, unicorn
4:30	book, reading
5:00	boy, clean up
5:30	Wild Krats, “animal grunt”
6:00	typing, “clicking”

**Table 3:** Communication modalities.

Mode of Communication	Beau		Carly		Maggie	
	Baseline	Intervention	Baseline	Intervention	Baseline	Intervention
Speech (single-word)	3.8	4.3	2.7	2.6	1.0	11.1
Speech (multi-word)	0.2	0.4	0.0	0.3	6.2	4.3
Sign/Gesture	1.0	1.4	0.3	0.4	1.8	0.5
Touch (no HS)	3.4	1.1	0.0	0.1	0.0	0.3
Touch label HS	0.0	10.0	0.0	13.8	0.0	9.0
Touch sound HS	0.0	2.3	0.0	5.0	0.0	4.3
Total	8.4	19.5	3	22.2	9	29.5
Total per min	2.2	2.6	0.6	2.8	1.8	3.9

The average number of symbolic communication acts in a turn and the use of communication modes, during baseline and intervention, per session.

## Data Availability

The raw data supporting the conclusions of this article will be made available by the authors on request. Additional information regarding study protocols is available in Patenaude (2025).

## Notes

1 Though Dominic met inclusion criteria, after baseline sessions, it was determined that this intervention was not appropriate for meeting his speech and language goals (i.e., increase social pragmatics, decrease pacing of speech). However, because he enjoyed the activity and the peer interaction, sessions continued with him intermittently, but no additional data was collected, and his data is not reported here.

2 This tablet is a product of Samsung Electronics Inc., 85 Challenger Rd, Ridgefield Park, NJ 07310, USA https://www.samsung.com/us/ (accessed on 1 February 2025).

3 Cocomelon is a YouTube channel of nursey rhymes (https://www.youtube.com/channel/UCbCmjCuTUZos6Inko4u57UQ (accessed on 1 February 2025).

4 Baby Shark is a children’s YouTube video (https://www.youtube.com/watch?v=XqZsoesa55w (accessed on 1 February 2025)).

5 Canva (www.canva.com) was used to create visual materials for this project, 3212 E. Cesar Chavez Street, Building 1, Suite 1300, Austin, TX 78702, USA.

6 Adobe (www.adobe.com) was used to create visual materials for this project, 345 Park Avenue, San Jose, California 95110, USA.

7 YouTube (www.youtube.com) was used to create visual materials for this project, 901 Cherry Ave. San Bruno, CA 94066, USA.

8 Scene and Heard Pro^TM^ is available from PRC-Saltillo, 1022 Heyl Rd, Wooster, OH 44691.

## Abbreviations

The following abbreviations are used in this manuscript:

AAC	Augmentative and Alternative Communication
VSD	Visual Scene Display
SLP	Speech and Language Pathologist
NDD	Neurodevelopmental Disability
CDI	Communicative Development Inventories
PLS-5	Preschool Language Scale-5th Edition
IOA	Inter-observer Agreement
